# Does Telecare Improve Interorganisational Collaboration?

**DOI:** 10.5334/ijic.2462

**Published:** 2016-11-24

**Authors:** Jannie Kristine Bang Christensen

**Affiliations:** Center of Organization, Management and Administration, Department of Sociology and Social Work, Aalborg University, Kroghstraede 7, 9220 Aalborg, Denmark

**Keywords:** telecare, home monitoring, interorganisational collaboration, intersectorial collaboration, horizontal integration, dependency structures

## Abstract

**Introduction:**

Previous studies have suggested that telecare can improve interorganisational collaboration within fragmented health care systems, yet this outcome has not been examined in a large-scale setting. This study explores the effects of a large-scale interorganisational telecare programme in Denmark based on home-monitoring on collaboration in a telecare network between municipalities, hospitals, and general practitioners.

**Methodology:**

Semi-structured interviews and observations of collaborating health professionals from the municipalities, hospitals, and general practitioners were undertaken and then repeated a year later. Collaboration was analysed both at the interorganisational network level and within each part of the network, including its interrelations.

**Results:**

Collaboration between municipalities and general practitioners was initially intensified as a result of implementing telecare, though this changed over time as the first start-up obstacles were overcome and the patients became more active in their treatment. Conversely, collaboration between *hospitals and municipalities* and *hospitals and general practitioners* was unaffected by telecare.

**Discussion:**

Changes in collaboration among municipal nurses, general practitioners, and hospital staff were related to dependency structures and municipalities’ newly gained central role in a telecare network. While the telecare network was initially characterised by asymmetrical dependency structures, these were partially equalised over time because of the municipalities’ new position in the network.

## Introduction

Health care systems in developed countries struggle with fragmentation of care, lack of coordination, and interorganisational collaboration [1,2,3]. Various political strategies have been developed and research undertaken to find solutions to each of these problems, yet fragmentation continues [2]. One attempt to address such problems has been through the innovation and implementation of digital tools that allow for fast and easy sharing of patient data [4,5]. For example, experimentation with new initiatives such as telecare is growing at a rapid pace throughout the majority of the world [6]. Telecare is a new health service that involves the use of technology within patients’ homes, such as home monitoring, safety monitoring, and information service technologies [7]. Certain of these technologies are already in broad use [8], though home monitoring has yet to be institutionalised within the conventional treatment of persons with chronic diseases (for an exception, see [9]). Various pilot studies of telecare show promising results, including enhancement of efficiency, improved quality of care, and better integration of care via the effective coordination of activities and collaboration between different health care providers [10,11,12,13].

As telecare services have yet to become fully mainstream, the majority of research in the field is based on pilot projects [4] and has focused on economical and clinical effects [13]. Few studies have investigated how telecare may contribute to integrate activities and collaboration between different health providers (e.g., [10,14]). Thus, the following research question was asked: *How does telecare affect interorganisational collaboration within a network of health care professionals from different organisations and political levels?* Contrary to prior studies, this study examines a large-scale, cross-sector Danish telecare programme involving more than 1,200 patients with chronic obstructive pulmonary disease (COPD) who receive remote home monitoring from an interorganisational telecare network of eleven municipalities, four hospitals, and 225 general practitioners (GPs). The study offers two substantial contributions. First, it deepens our empirical knowledge of telecare in a complex, large-scale setting with multiple health care organisations. Second, it provides a nuanced understanding of how telecare reconfigures interorganisational networks in terms of interorganisational collaboration, dependency, and power structures.

## Context: The Danish Health Care System

The Danish health care system is a mainly public system based on general taxation. The system is characterised by rather strong regulation from the state, and is managed politically at the state, regional, and municipality levels. The health care system is organised into primary and secondary health sectors. Primary health care services are mainly provided by two separate actors: self-employed GPs (family doctors) and municipalities (the local political level). GPs act as gatekeepers to the health care system, as the majority of access to hospital treatment (except for emergency visits) and municipal health services requires referrals from GPs. Municipalities provide preventive care, home care, and rehabilitation. Secondary health care services are provided by hospitals, which are led by regions (the regional political level). Hospitals perform specialised treatment, both during hospitalisations and as a part of outpatient clinic services [15]. Even though the Danish health care system is perceived to be one of the most integrated systems in Europe, it nonetheless struggles with fragmentation challenges [3]. Such fragmentation creates extensive demand for the integration of activities between the three main health providers (municipalities, hospitals, and GPs), especially concerning patients with chronic conditions [10].

## Case

**Figure 1:**
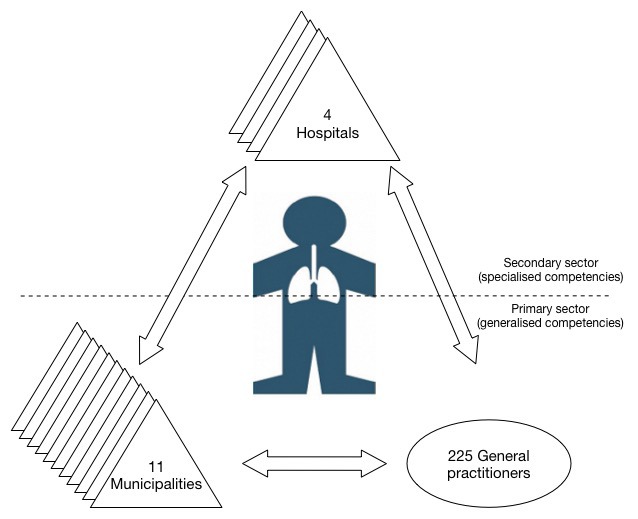
Interorganisational telecare network.

The paper is based on a qualitative case study of *TeleCare North* [16], the largest Danish telecare programme. This programme is rather distinct both in Denmark and internationally because it involves interorganisational collaboration between health care actors in the primary and secondary health sectors. Figure 1 depicts the actors in the telecare network of the programme.

The aim of the programme is to improve collaboration between different health care providers, for example, by providing shared access to the same monitoring database. Furthermore, savings in terms of hospital (re)admissions and improved quality of life are expected outcomes of the programme [16].

*TeleCare North* focuses on the home monitoring of patients with COPD who live in the northern part of Denmark. These COPD patients self-measure oxygen level, blood pressure, pulse, and weight, and answer questions about their symptoms. This data is then sent to a shared monitoring database that allows not only GPs to access and monitor patients’ data, but also health professionals from municipal health centres, municipal district nurse units, hospital wards, and hospital outpatient clinics. Conventionally, various groups of health care providers use their own electronic documentation systems that are not accessible to others outside their organisation. The *TeleCare North* programme is managed by a steering group with representatives from municipalities, hospitals, GPs, and other relevant actors (e.g., patient unions and the National Agency for Digitisation), with a composition that reflects the interorganisational setup of the programme. At the administrative level, the programme’s steering group facilitates interorganisational collaboration [17]. At the operational level, formal agreements between the municipalities, hospitals, and GPs assign roles and tasks to different municipalities, hospitals, and GPs. Municipalities are responsible for monitoring patients in a stable COPD course (which characterises the majority of patients in the programme), while hospitals are responsible for monitoring the most severe COPD patients. GPs are responsible for referring patients to the programme, and have an on-going responsibility to adjust each patient’s measurements, such as concerning acceptable levels of oxygen in the blood. GPs serve much like a form of medical consultant to municipal nurses.

## Conceptual Framework

To understand how telecare can integrate care across the different health care providers, it is necessary to understand how integration can be obtained. According to Axelsson and Axelsson (2006), integration can be divided into vertical integration and horizontal integration. Vertical integration denotes integration between organisations or organisational units at different hierarchical levels, whereas horizontal integration denotes integration at the same hierarchical level. Collaboration involves a high degree of horizontal integration and a low degree of vertical integration. Collaboration can be difficult to achieve, as it often relies on voluntary agreements and mutual adjustments between organisations in the absence of a common hierarchical structure [17,18]. Alternatively, cooperation involves both a high degree of vertical integration and a high degree of horizontal integration. Cooperation combines hierarchical control mechanisms with greater voluntary network collaboration in a complex matrix organisation [19], requiring a kind of common hierarchical system or formal agreements in interorganisational contexts.

Collaborative processes and interorganisational relations often take place within a network structure. Interorganisational networks emerge through the repeated interactions of organisational actors from different organisations, and are the result of different kinds of interdependencies between network organisations in terms of solving tasks or achieving certain goals [17,20,21]. By entering or forming networks, organisations gain access to new resources, such as information, competencies, knowledge, and money, which make it possible to solve tasks that they could not otherwise have handled by themselves [2,17,20,21,22].

Numerous kinds of network types exists (e.g. joint ventures, strategic alliances etc.) [23] but a certain type is of interest to this study because it denotes relations between different kind of organisations that collaborate to reach a shared goal: a systemic network. Such networks consist of different organisations with complementary competencies, services, or products that collaborate to solve a shared task in an interorganisational context. Completing shared goals requires the functional differentiation of roles, responsibilities, and tasks, as well as horizontal processes of collaboration and the integration of activities in a network [10,17]. Thus, organisations are highly dependent on each other to solve shared tasks. Due to such dependency, tensions and conflicts may arise between the organisations, especially when one actor is more dependent on another. According to resource dependency theory, dependent actors have a weaker position in a network and may attempt to countervail asymmetrical dependency structures by forming coalitions or searching for alternative collaborators [20]. However, such attempts do not often go unnoticed by the more powerful actors in a network, who typically counteract to remain in a powerful position [24,25].

Interorganisational relations and collaboration processes are thus dynamic, loaded with moves and countermoves to achieve the most advantageous position within a network. Furthermore, negotiations and power struggles concerning specific domains and the division of labour serve to (re)define, and sometimes blur, the boundaries between professionals and organisations within a network. This is especially evident in health care systems that include professional bureaucracies which rely on highly trained professionals (e.g. doctors), and are characterized by a high degree of functional specialization and decentralized decision-making structure [26,27,28]. According to Abbott (1988) the implementation of new tasks and technologies can create disturbances in existing power relations between professions and within systems of professions. Within systemic networks that consist of professional bureaucracies, tensions and power struggles should therefore be anticipated.

Based on this conceptual framework, this study investigates the horizontal collaboration processes and dependency structures at the operational level among various health care professionals across different municipalities, hospitals, and GPs within a systemic network where tasks are predominantly mandated through formal agreements. This network is characterised by a high degree of functional specialisation and complexity, where conflicts, tensions, and power struggles among the different health professionals and the organisations are very likely to be part of collaboration processes.

## Methodology

### Data Collection

This study uses qualitative methods to collect data concerning the effects of telecare on collaboration within an interorganisational telecare network. Nurses from various municipalities’ health centres, district nursing units, hospital wards, and outpatient clinics, as well as physicians from hospital wards and GPs, were interviewed and observed in this study. The interviews were repeated one year after the first were conducted, for a total of 28 semi-structured interviews and 10 hours of observation. The participants were recruited through local project managers in the organisations in the programme, except for the GPs, who were recruited through a direct contact. As a result, five municipal nurses, two hospital nurses, two lung physicians, and six GPs participated in this study.

The study’s interviews were based on an interview guide that includes theoretical concepts about collaboration (in terms of information flow, knowledge exchange, and boundary spanning activities), interorganisational relations (the interactions, strengths, and reciprocity between relationships), dependency structure, interorganisational and interprofessional conflicts, and descriptive themes concerning the division of labour (roles and function), task changes, and the integration of telecare tasks in existing work practices. The interviews, a combination of face-to-face interviews and telephone interviews, lasted 30–70 minutes, and were transcribed in full.

Five of the interviewed nurses were observed as they performed telecare activities. Three of the observed nurses came from municipalities, with the rest coming from a hospital setting. The observations focused on how the nurses assessed patient data and communicated with patients and other health care professionals, such as GPs, municipal, and hospital staff when assessing such data. During the observations, the nurses often spoke of their frustrations related to collaboration with other actors, as well as difficulties using the telecare database. Furthermore, they often articulated tacit knowledge and practices in relation to telecare that were not mentioned in the interviews [27]. Such information was documented in extensive field notes written immediately following the observations. Most of the conversations during the observations further expanded upon certain topics of the interview. However, the observations also revealed new perspectives and topics that were not part of the interviews. For instance, while a lack of trust between municipal and hospital nurses was not mentioned in the interviews, it was revealed in the observations.

The data collection took place six and eighteen months after the first patients were enrolled in the telecare programme, respectively. Both interviews and observations were conducted by the same researcher (the author), who had been studying the telecare programme closely since its inception two years prior.

### Analytical Approach

The study of interorganisational networks and how a new telecare programme may improve collaboration among networked health care providers was done by switching analytical lenses of zooming in on each organisational part of the network and zooming out to the network as a whole at large [29]. Zooming in on the organisational level made it possible to investigate how each part in the network utilises and perceives collaboration with the other parts of the network. Conversely, zooming out to the network level enabled analysis of the network’s goals and outcomes as a whole, as well as how the interorganisational network changed as a result of implementing telecare. In addition, this analysis illuminated the effects of the new telecare programme on interorganisational collaboration. The combination of these two analytical lenses served to gain knowledge as to how telecare may be used to improve collaboration among different health providers across multiple professions, organisations, and political levels.

The analysis was performed in four stages. First, the transcribed interviews and field notes were thematically coded [30] using the qualitative software programme NVivo. The codes were partly constructed from theoretical concepts in the above-mentioned interview guide, and partly emerged from the empirical data (e.g., lack of trust). Second, the data were coded in terms of each organisation in the network and the dyadic relations between them, including changes in work routines, roles, and interorganisational relations. Third, the analysis focused on the aggregate level of the network, focusing on changes of position within the network, network outcomes, interorganisational dynamics, interrelatedness between dyadic relations, and interorganisational collaboration. Finally, the findings of this study were presented to local project managers within the municipalities and hospitals, the steering group of the telecare programme, and other practitioners in the field. During these presentations, the results were discussed and validated as widely recognised among the practitioners.

## Results

The results of this study are presented in three sections. In the first section, the findings concerning interorganisational collaboration between municipalities and GPs within the primary health sector are presented by zooming in on the dyadic relations and collaborative efforts between these organisations. The second section offers findings concerning the collaboration between primary health sector (GPs and municipalities) figures and hospitals in the secondary health sector. The third section identifies changes of interorganisational collaboration within the telecare network between the two data collections. Based on these results, the findings concerning the broader telecare network and implications for interorganisational collaboration among the three different health providers in the network are given.

### Collaboration among Municipal Nurses and GPs in the Primary Health Sector

The analysis of each organisational part of the telecare network revealed that only municipal nurses experienced significant changes in their work after the implementation of telecare, which in turn affected their collaboration with GPs. Traditionally, municipal nurses are generalists that lack training within a specialised field. In the telecare network, the majority of smaller municipalities had only generalist nurses, whereas the larger municipalities had specialised nurses. Half of the interviewed municipal nurses did not have specialised COPD competencies. The study’s observations revealed that municipal nurses were struggling when assessing patient data, as this new task required specialised, in-depth knowledge about COPD. Telecare was found to have forced the municipal nurses into specialist roles formerly belonging to hospital nurses.

The new requirements of these specialist roles affected the nurses’ collaboration with GPs, as they required increased support from GPs for the legitimacy of their data assessment. The result was more intense collaboration between municipal nurses and GPs due to the significant increase of queries from municipal nurses. Moreover, collaboration itself became more professional because, through the use of telecare, the inquiries of the municipal nurses were more precisely formulated and supported by comprehensive knowledge and information regarding patients’ conditions. One GP expressed how collaboration was professionalized as a result of telecare of the collaboration as follows:

“The municipal nurses can now deliver certain interesting observations of patients which I find useful. So, yes, telecare supports our collaboration.”

The positive perception of collaboration after the implementation of telecare also resonated in the municipalities, as explained by a municipal nurse:

“Now I communicate more and better with the GPs because our communication has more substance than before. I get more professional inputs, which I would not have gotten from another nurse. So, yeah, I really appreciate it.”

In several cases, intensified collaboration was recognised as a way of increasing quality of treatment for the involved COPD patients.

Collaborative efforts in relation to telecare were initiated solely by the municipal nurses, who were highly dependent on the GPs’ medical expertise. From the GPs’ perspective, they could solve tasks independently of the nurses, and furthermore, felt no obligation to collaborate with the nurses. This asymmetrical dependency left the municipal nurses in a vulnerable position, leading to frustrations with GPs that were unwilling to collaborate. Despite the seemingly subordinate position of the municipal nurses, however, they were able to challenge the GPs’ position and authority in the decision-making process due to their newly gained knowledge about COPD and the patients’ conditions which was gained through telecare.

Both the nurses and the GPs articulated underlying issues of interprofessional tension in the interviews and observations. The GPs expressed that the municipal nurses were controlling their work and questioning their decisions about the treatment of the COPD patients. Consequently, they felt that the municipal nurses were infringing upon their professional domain. As for the municipal nurses, they expressed a similar sentiment, though in a slightly different way. Some of the nurses had experiences with GPs that suddenly became hostile and very protective of their status as clinical decision-makers. One of the nurses explained this hostility:

“I suggested another self-treatment plan to one of the GPs and this annoyed the GP. She wouldn’t comply with my suggestion because, she said, ‘I have the clinical knowledge and expertise in this field. I’m in charge and I decide how this patient is treated’. It was like she wanted to put me in my place.”

The majority of the municipal nurses also spoke about how their new knowledge gave them greater influence in relation to the GPs in terms of treatment and in the clinical decision-making process. Regardless of these underlying issues and asymmetrical dependency relations, however, telecare supported the interorganisational collaboration between municipal nurses and GPs within the primary health sector by making the collaboration more professional.

### Collaboration between the Primary and Secondary Health Sectors

In general, collaboration facilitated by telecare services among health care professionals from hospitals in the secondary health sector and the municipalities and GPs of the primary health sector was very restricted. The interviewed health care professionals from each of these areas characterised cross-sector collaboration as weak or non-existent. One hospital nurse discussed the weak ties between her and the GPs:

“I haven’t been collaborating with the GPs at all in relation to telecare. (…) Actually, I don’t find it necessary to collaborate more extensively with them. If they refer a patient to hospital treatment, well, then the referral is enough communication for us. What else do we need to collaborate about? So, our collaboration with the GPs can be characterised as non-existent.”

No interdependencies between hospital nurses and GPs were acknowledged by all interviewees. Similarly, the lung physicians, for example, did not express any dependency on the GPs or the increased need for collaboration. In line with this statement from the hospital nursenearly every GP was surprised to hear that the hospitals were a part of this programme even though it had been implemented for nearly six months at the time they were first interviewed. This clearly exemplified the non-existent collaboration between the hospitals and GPs. Similar to the hospital staff, none of the GPs expressed a need for greater or extended collaboration.

At the municipalities, the need for interorganisational collaboration with the hospitals was more pronounced. The municipal nurses expected better information flow and knowledge exchange with hospital staff to be one of the goals of telecare. However, these expectations were not met, as one of the municipal nurses explained:

“We had one patient who was hospitalised. During his hospitalisation they changed his medicine and oxygen treatment. However, we were not notified at all, even though the hospital staff and his GP knew he was part of this telecare programme [and that we monitor his data, ed.] (…) Afterwards, we talked to the patient, and he assumed we knew about the changed medication and oxygen treatment, but we didn’t.”

This lack of integration in terms of knowledge and activities between municipalities and hospitals created fragmented care for patients in two ways. First, missing information related to hospitalisation meant that municipal nurses lost a degree of their authority in terms of knowledge and were not able to build a treatment alliance with the hospital staff. Instead, communication across the sectors appeared incoherent. Therefore, telecare did not mediate a shared understanding or better information flow between the different health care providers from the municipalities and hospitals. Second, hospital actors did not integrate municipal nurses’ or GPs’ knowledge about the COPD patients into their work activities. For example, regular check-ups at the outpatient clinic were held as usual without the integration of telecare data or observations from the municipal nurses. The frequency of the check-ups was not changed regardless of whether or not the municipal nurses deemed patients to be on a stable COPD course without exacerbations, which reflected the limited collaborative efforts between the municipalities and hospitals. Hence, the integration of knowledge in the patients’ courses and changes in behaviour was limited when relying solely on the voluntary behaviours and good-will of the collaborators.

One episode, however, within a municipal nursing district unit, serves as an example of successful collaboration, as the following sequence from the study’s observation notes illustrates:

“The municipal nurse calls a patient because his oxygen level is very low. They talk about his latest hospitalisation and how the physician at the lung ward recommends oxygen treatment. The municipal nurse supports this recommendation and the patient seems more convinced.”

This example illustrates how the two actors successfully collaborated across the primary and secondary sectors in an alliance so as to convince a patient about starting oxygen treatment. As a result, the information and recommendations from the two sectors were coherent and integrated for the patient. In this case, telecare created an opportunity to collaborate and mediate a shared understanding of the patient’s treatment.

Even though collaboration in most cases was close to non-existent, conflicts between municipal and hospital nurses were nonetheless identified in the study’s interviews and observations. These conflicts concerned distrust of each other between the nurses. Certain patients were monitored at different times by both the municipalities and the hospitals. The majority of the nurses, regardless of their organisational affiliation, checked up on their patients when they were monitored by the other party, even though they were not supposed to, which led to suspicions concerning how the other party was reacting to patient data. One of the municipal nurses expressed this issue in the following way:

“Collaboration with the hospital has not been very successful. We have a patient who is currently being monitored by the hospital. I am curious, so I still check his data. I discovered that the hospital doesn’t really react to bad vital signs from him. So I don’t think the collaboration actually works.”

This nurse was not able to access the hospital’s electronic medical record so as to see how the hospital nurses were reacting to the patient’s measurements, but could only see the patient’s basic data. Similar situations were observed in the hospitals. In the study’s observations, it was evident that the counterpart (either the municipal or hospital nurses) did in fact react and offer treatment based on poor measurements, but this was not noted in the monitoring database in the telecare programme. As the health professionals only documented their actions and decision-making processes in the medical records of their own organisations, the sharing of knowledge was highly restricted. Consequently, the inability to gain insight into other institutions decision-making processes was a barrier to interorganisational collaboration.

### Changes in Interorganisational Collaboration

As demonstrated, telecare predominantly affected collaboration in the primary sector between municipalities and GPs. However, networks are unstable entities that fluctuate and change according to different network dynamics [31], which was found to be the case in terms of horizontal collaboration processes in a year follow-up examination. Two main changes were identified. The first concerned collaboration between GPs and municipal nurses. Between the first and the second interview round, collaboration was found to have decreased. One hypothesis for this was that the need for collaboration simply decreased after initial challenges with telecare and adjustment of the programme were overcome. However, this was not altogether true. In certain cases, decreased collaboration was a result of telecare being utilised mainly as a mono-organisational service, with municipal nurses solving telecare tasks independently of GPs or hospitals. Interorganisational collaboration was thus in these cases almost non-existent. GPs were detached from the telecare services and no longer had any interactions with them. In other cases, the positive dynamic between the municipal nurses and GPs found in the study’s first interview round was enforced, namely, with regard to the quality and professionalism of the municipal nurses’ inquiries to GPs. One GP explained this on-going positive dynamic as follows:

“Collaboration with the municipal nurses is much better. It is more relevant; the questions from the nurses, who have all of this information from the patients’ self-monitoring, have become much more relevant compared to the beginning of the programme.”

In these cases, there seemed to be greater mutual acknowledgement of interdependency and complementarity between the GPs and the nurses since the first interviews, with dependency relations appearing less asymmetrical, even though the nurses were still more dependent on the GPs than vice versa. The second change was related to the patients’ role, as they had gained a more active role in their treatment and were more empowered to start treatment themselves according to their self-treatment plans. As a result, collaboration between municipal nurses and GPs became more indirect and mediated through the patients themselves. A municipal nurse explained this change of empowerment as follows:

“In the beginning of the project, the patients disclaimed responsibility for their disease. They expected me to contact their GP when they felt bad. Now, however, most of them have taken responsibility for their disease; they are in charge now.”

One GP elaborated on the indirect collaboration created by patients, who serve as links between different health providers:

“The patients are the link between the municipal nurses and me. They contact me because their municipal nurse told them to.”

Several GPs, however, stated that patients still perceive them as the medical authorities, and that the latter informs them each time they start self-treatment, even though the GPs do not require this information. Thus, it appears that the GPs’ role as the medical authority remains intact despite the new central role of municipal nurses in patient courses. Despite these continuing changes, the amount of collaboration between the primary and secondary health sectors remained unchanged and almost non-existent.

## Discussion

One of the main objectives with *TeleCare North* was to improve collaboration by developing and implementing an interorganisational telecare service among the three main health providers in Denmark (municipalities, GPs, and hospitals). The findings of this study reveal that telecare affected interorganisational collaboration to varying degrees, and that these degrees further changed over time. The analysed dyadic relations between municipalities and GPs in the primary sector and between the primary and secondary sectors, however, cannot be understood without taking into account other relations and dynamics in the network. Thus, the findings related to the dyadic relations at the broader network level will here be discussed.

One of the basic aspects of systemic networks is that organisations are mutually dependent on each other to solve a joint task [17]. Interorganisational relations are interconnected in a complex web, with changes to certain relations affecting other relations in the network. Therefore, when collaboration between GPs and municipal nurses is enforced, it both affects and is affected by the interorganisational relations between *GPs and hospitals* and *hospitals and municipalities*. Interconnectedness was witnessed in the network in the following ways. Strong ties between GPs and municipal nurses were often associated with weak or non-existing collaboration with hospitals in the telecare network. Stronger collaboration and enhanced competencies in the primary sector appeared to supplement demand for hospital services and expertise when delivering daily telecare services. As a result, the hospitals’ role and functions in the telecare network were nearly invisible to the other actors within it, which the following quote from a hospital nurse illustrates:

“It is my impression that the municipal nurses are skilled when handling the COPD patients. They don’t need our expertise. Before [the telecare programme, ed.] we perceived our self as *the experts*, and of course we are still the experts in some aspects, but when it comes to COPD, we are quite equal with the municipal nurses, who assess the patients’ data.”

Despite their near invisibility to the other players in the network, no counteractions were taken by the hospitals to re-establish the dependency structures that favoured their powerful position as COPD experts.

In other cases where collaboration between municipal nurses and GPs was weak or non-existent, more informal, ad hoc collaboration between municipal nurses and hospital staff emerged, with GPs distanced within the telecare network. Traditionally, collaboration between municipal nurses and hospitals was mediated by GPs, who referred patients to hospitals or municipal health services. However, when the GPs refused to collaborate and mediate the link between the hospitals and municipalities the municipal nurses found alternative strategies to collaborate directly with hospitals when GPs refused to participate and serve as mediators. A municipal nurse commented on this issue as follows:

“We asked the GP about a self-treatment plan, but he refused to take it, so instead we contacted the lung physician at the hospital, who made a more comprehensive treatment plan (…). So, we find our loopholes [when the GPs refuse to collaborate, ed.].”

The above comment reflects the asymmetrical dependency structures of the telecare network and how they force municipal nurses to initiate and maintain collaboration with various medical experts (GPs, hospital nurses, or doctors). Such unequal dependency structures speak to how more dependent organisations (in this case, the municipalities) are left in a vulnerable position in terms of support and ability to react properly on poor measurements. However, as has been shown in this study, it is nonetheless possible for dependent organisations to work their way around certain obstacles in a network and build interorganisational relations to fulfil their needs.

Based on the findings of this study, it is important it is to take into account power and dependency structures when studying networks. These structures have often been omitted in studies on networks, as mutual dependency has been assumed to equalise power asymmetries [32]. Indeed, such power and dependency structures are not stable, but fluctuate and change according to network dynamics and changes in network organisations and broader contexts [24,31,33]. Fluctuation and changes in power and dependency structures was evident in the telecare network when municipal nurses became less dependent on medical expertise as they became accustomed to telecare tasks and gained more specialised knowledge concerning COPD and their patients. Consequently, the dependency and power structures in the telecare network changed, and the three health providers could act more independently in solving telecare tasks. However, with this came the risk of losing the incentive to collaborate.

Each of the actors in the network was not able to reach the shared network goals alone. For example, the network set the goal of reducing ordinary check-ups at the hospitals and among GPs. To fulfil this goal, both hospitals and GPs were dependent on municipal nurses and their assessments of patients’ conditions. However, the hospital staff and GPs continued to work independently of the municipal nurses, and thus the network goal was not reached. To achieve this goal, a greater balance between autonomy and dependency in the network was required, which should be developed and maintained through the effective management of horizontal network processes [17,34]. The findings of this study further suggest that the integration of activities in the telecare network must be achieved beyond mere collaboration. For example, it may be beneficial to focus on vertical integration through hierarchical mechanisms. That is, cooperation [18] – which involves a high degree of hierarchical control mechanisms, voluntary agreements, and mutual adjustments between organisations – may be a more useful method for developing complex health services that cross organisational boundaries.

## Methodological Considerations

This study followed a qualitative case study design. Throughout the study, rich descriptions of the organisational settings and contexts allow the findings of the study to be transferred to similar settings, as well as be generalised for further analyses. The internal validity and credibility of the results were gained through the presentation to and validation of the findings by practitioners in the telecare programme. Even though the results were controversial (as they revealed that network goals were not achieved), the different practitioners confirmed the findings within their own organisations. Moreover, the researcher’s insight into the field enhanced the credibility of the findings [35].

The analytical choice to divide the network into dyadic relations may be perceived as a limitation of the study. The decision was made to decrease the complexity and comprehensiveness of full network analysis. Though, dividing the network into dyadic relations does not offer a full analysis at the network level [22]. Analysing networks at the network level, however, was beyond the scope of this study. Consequently, a full explanation of the network’s dynamics is not offered in this study. Instead, the dyadic relationships and their interconnectedness are investigated and discussed in relation to network goals and dynamics.

## Conclusion

The implementation of telecare was found to affect interorganisational collaboration between municipalities, hospitals, and GPs to varying degrees. The changes identified in this study with regard to interorganisational relations were related to structural properties, power, and dependency structures in the telecare network. The telecare network was centralised, with the municipalities serving as its central organisations. This central position gave the former power, and thus the municipal nurses had increased influence on COPD treatment, which challenged the medical authority of GPs, as well as generated intra-professional conflicts between the hospital and municipal nurses. The municipalities were put in a vulnerable position, as they were significantly more dependent on the medical expertise of GPs or hospital staff than the other way around. This dependency initially instigated intensified collaboration among municipal nurses and GPs. When collaboration with the GPs was impossible or difficult to establish, the municipal nurses found alternative strategies for receiving medical expertise from hospital staff. Otherwise, the hospitals were nearly invisible to the other actors in the telecare network. The dependency structures, however, changed during the period that the telecare network was studied. The municipalities became less dependent on medical expertise as their experiences and knowledge about monitoring COPD patients grew. Accordingly, municipal nurses’ collaboration with the GPs was less intense. However, both the GPs and municipal nurses characterised their collaboration as more professional and relevant as a result of telecare use, and that such professionalization had in certain cases been reinforced over time. At the same time, the telecare programme also led to interprofessional power struggles, as the municipal nurses challenged the GPs autonomy and positions as medical authorities. This study illustrates how networks fluctuate and change according to internal network dynamics and external dynamics. To improve or change interorganisational relations, continual effort and attention must be given to the power and dependency structures of networks and their interrelated dynamics.
